# Psychogenic Purpura Successfully Treated with Antidepressant Therapy

**DOI:** 10.4274/tjh.2016.0505

**Published:** 2017-08-02

**Authors:** Şeyda Çelik-Göksoy, Ayşe Kılınçaslan, İlyas Kaya

**Affiliations:** 1 İstanbul University Faculty of Medicine, Department of Child and Adolescent Psychiatry, İstanbul, Turkey

**Keywords:** Psychogenic purpura, Gardner-Diamond syndrome, Antidepressant, adolescent

## To The Editor,

Psychogenic purpura (PP) or Gardner-Diamond syndrome is a very rare condition characterized by spontaneous, recurrent, and painful edematous skin lesions progressing to ecchymoses [1]. Bleeding from the nose, gastrointestinal organs, kidneys, and uterus has also been reported [2]. PP is commonly regarded as an autoimmune vasculopathy with sensitization to phosphatidylserine, a component of erythrocyte membranes [3]. Development of the lesions generally follows minor physical trauma and/or emotional distress, and they are often accompanied by certain psychiatric conditions [4]. However, it is still unclear how stress influences the physiological processes and changes the immune reactivity so that organisms react with the formation of erythrocyte autoantibodies [1]. Here we present a case associated with depression and sexual abuse.

**Figure 1:**
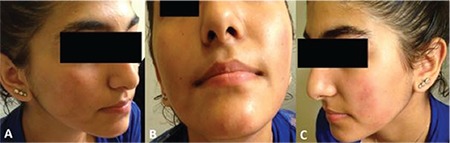
Ecchymotic lesions at the time of referral (4^th^ day).

A 15-year-old female was referred with complaints of recurrent ecchymotic bruising over her cheeks and chin (Figure 1). The lesions started with a burning sensation, severe right-sided hemi-headache, and nausea. Afterwards, reddish discoloration progressing to ecchymoses within a couple of hours appeared. The lesions became less painful and disappeared spontaneously within a week.

The painful ecchymotic bruises started 5 years ago, after sexual abuse by a neighbor involving cuddling, kissing, and fondling. It occurred once, it was kept secret, and the family moved to a new house. The lesions occurred 3 months after the assault and they were not associated with physical trauma or drug use. They recurred periodically at intervals of 3-4 months, but sometimes more frequently (i.e. once in 15-20 days). There was no personal or family history of bleeding. The systemic examination was unremarkable. Exhaustive laboratory investigations and hematological examinations, including complete blood count with differential liver function, prothrombin time, activated partial thromboplastin time, fibrinogen, von Willebrand factor, and platelet aggregation tests yielded results within normal limits. A direct Coombs test was negative and erythrocyte and platelet morphologies, platelet clustering, and complete urinalysis were normal. Rheumatological examinations, including erythrocyte sedimentation rate, rheumatoid factor, C-reactive protein, and lupus anticoagulant, were within normal limits. A skin biopsy specimen of an ecchymotic plaque showed extravasated red blood cells throughout the dermis with no inflammatory infiltration.

In the psychiatric examination, she reported depressive mood, anhedonia, hopelessness, and concentration problems. She disclosed being sexually abused (i.e. kissing and fondling with no sexual intercourse) by her father from the age of 12. She had mild suicidal thoughts but described no plans or prior attempts. She reported her worries about her mother’s medical problems and the future. She obtained a score of 28 on the Beck Depression Inventory (BDI) and 41 on the self-report Screen for Child Anxiety Related Disorders (SCARED). The diagnosis of PP was confirmed upon seeing a typical ecchymotic lesion 7 h after the intradermal injection of autologous erythrocytes [4].

**Figure 2:**
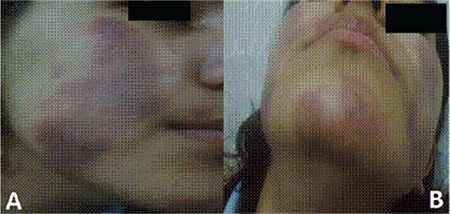
Recurrence of the lesions after cessation of the antidepressant (early on the 2^nd^ day).

An investigation was launched by Child Protection Services and she was assigned to stay in a residential unit for 2 weeks. Meanwhile, her father moved away and she returned home to live with the rest of her family under the supervision of Child Protection Services. Antidepressant treatment with escitalopram at 5 mg/day was started and increased to 10 mg/day. Her suicidal thoughts and depressive symptoms decreased. She started sports and drawing. She stopped her medication in the fifth month. However, another episode occurred after 4 weeks (Figure 2). This time, previous symptoms were accompanied by hemoptysis. Laboratory investigations, bronchoscopy, and thoracic computerized tomography revealed no abnormality and there was no obvious stressor to trigger the episode. However, psychiatric examination revealed subclinical depressive symptoms. She used escitalopram for 2 years, which was stopped after complete resolution of her depression (BDI: 8, SCARED: 14). She had no other episodes, neither under antidepressant therapy nor in the 12-month drug-free period.

Cases presented in the literature commonly give information about the clinical features and differential diagnosis of PP. However, the treatment and follow-up of cases are rarely described [5]. This adolescent had many typical episodes of PP associated with emotional distress and depression. The cessation of the sexual abuse and antidepressant therapy with escitalopram treated both the PP and depression. This case was presented to highlight the importance of psychiatric treatment in such cases.
